# Preventing Recurrent Otitis Media in Children Aged 2–7 Years: A Cross-Sectional Evaluation of Serum Vitamin D as a Modifiable Factor

**DOI:** 10.3390/diagnostics15050519

**Published:** 2025-02-20

**Authors:** Alexia Manole, Lavinia Florica Mărcuț, Răzvan Cârciumaru, Felicia Manole

**Affiliations:** 1 Faculty of Medicine and Pharmacy, University of Oradea, 410087 Oradea, Romania; manole.alexia@student.uoradea.ro; 2 Department of Surgical Disciplines, Faculty of Medicine and Pharmacy, University of Oradea, 410087 Oradea, Romania; marcut.laviniaflorica@didactic.uoradea.ro; 3 Doctoral School of Sociology, Faculty of Social and Human Sciences, University of Oradea, 410087 Oradea, Romania; 4 ENT Department, Faculty of Medicine and Pharmacy, University of Oradea, 410087 Oradea, Romania; fmanole@uoradea.ro

**Keywords:** vitamin D, otitis media, recurrence prevention, children, vitamin D deficiency, vitamin D insufficiency, immune function, pediatric otolaryngology

## Abstract

**Background/Objectives**: Otitis media (OM) is a common pediatric condition that significantly impacts hearing, language development, and quality of life. Emerging evidence suggests that vitamin D plays a crucial role in immune regulation and that deficiency may predispose children to recurrent OM. This study aimed to evaluate whether low serum vitamin D levels are associated with increased incidence and severity of OM in children aged 2–7 years. **Methods**: A cross-sectional study was conducted at a pediatric otolaryngology clinic in northwest Romania between November 2023 and April 2024. A total of 118 children were enrolled, including 87 children with documented episodes of OM within the preceding six months and 31 quasi-controls without recent OM episodes. Participants were stratified into three age groups (2–3, 4–5, and 6–7 years) and classified into clinical subtypes of OM (Acute Suppurative Otitis Media, Serous Otitis Media, and Acute Congestive Otitis Media). Serum 25-hydroxyvitamin D levels were measured using a chemiluminescence immunoassay and categorized as deficient (≤20 ng/mL), insufficient (21–29 ng/mL), or sufficient (≥30 ng/mL). Statistical analyses included one-way ANOVA with post-hoc tests, chi-square tests, linear regression, logistic regression, and Poisson regression. **Results**: Children with OM exhibited significantly lower vitamin D levels compared to controls, with the most pronounced deficiency observed in the Acute Suppurative OM group. One-way ANOVA revealed significant differences among the groups (F(3,114) = 82.30, *p* < 0.001), and linear regression demonstrated a strong inverse correlation between vitamin D levels and the frequency of OM episodes (r = −0.793, adjusted R^2^ = 0.63, *p* < 0.001). Logistic regression indicated that vitamin D insufficiency significantly increased the odds of OM (OR ≈ 120.74, *p* < 0.001), while Poisson regression showed incidence rate ratios of 13.62 for deficient and 10.47 for insufficient vitamin D status (*p* < 0.001). **Conclusions**: The findings indicate that low serum vitamin D levels are significantly associated with an increased risk and frequency of otitis media in preschool-aged children. These results support the role of vitamin D deficiency as an independent, modifiable risk factor for recurrent OM, suggesting that vitamin D screening and supplementation could be beneficial in reducing the clinical and economic burden of this condition.

## 1. Introduction

Otitis media (OM) is one of the most prevalent pediatric conditions worldwide, affecting millions of children and contributing significantly to healthcare burdens through frequent medical consultations, antibiotic prescriptions, and even long-term complications such as hearing loss and speech delays [1,2,3,4,5]. Characterized by inflammation of the middle ear, OM is not a uniform disease but rather a spectrum of clinical entities with varying presentations and outcomes. The condition has been traditionally classified into distinct subtypes, each of which reflects different underlying pathophysiological processes.

Acute Suppurative Otitis Media (ASOM) is typically characterized by the presence of a perforated tympanic membrane accompanied by purulent discharge, indicating a severe inflammatory response to bacterial invasion [6,7]. In contrast, Serous Otitis Media (SOM) involves the accumulation of a sterile effusion behind an intact tympanic membrane, which, although less dramatic in its presentation, can lead to conductive hearing loss and subtle yet significant developmental delays [8,9]. Meanwhile, Acute Congestive Otitis Media (ACOM) presents with marked inflammation and pain without the perforation of the tympanic membrane, representing a milder form of the disease [8,9,10]. In many clinical settings, including our local practice, these terminologies are commonly used to differentiate disease presentations even if some international studies favor alternative nomenclature. Recognizing these differences is crucial for both diagnosis and treatment, as the management strategies and prognosis may vary among the subtypes.

Beyond the clinical distinctions of OM, an increasing body of evidence has focused on the role of modifiable factors that might influence the susceptibility to and severity of OM. Among these, vitamin D has emerged as a key player. Traditionally known for its essential role in calcium and phosphate homeostasis, vitamin D also plays a pivotal role in the regulation of the immune system [11,12]. It enhances innate immunity by promoting the production of antimicrobial peptides such as cathelicidin and defensins, which serve as first-line defenders against invading pathogens [13,14]. Additionally, vitamin D modulates adaptive immune responses by balancing pro-inflammatory and anti-inflammatory cytokines, thereby potentially reducing tissue damage during infections [14,15]. Several studies have reported a strong association between vitamin D deficiency and an increased incidence of respiratory tract infections, including OM [16].

Recent advances in food biochemistry underscore that dietary factors extend well beyond basic nutrition, influencing the key biochemical pathways involved in disease prevention. Studies have demonstrated that bioactive compounds from food can modulate the metabolic and signaling pathways critical for the control and prevention of nervous system diseases [17,18,19,20,21]. Although some of them focus on neurological outcomes, the underlying principles suggest that a comprehensive nutritional strategy—one that includes optimal vitamin D levels along with other beneficial dietary components—could enhance immune function and reduce inflammation. This holistic approach may have significant implications for the prevention of infections such as OM, highlighting the need to consider the broader role of nutrition in maintaining overall health.

The importance of vitamin D as a modifiable factor is further highlighted by previous research demonstrating that suboptimal vitamin D status may lead not only to a higher risk of infections but also to increased healthcare costs and broader public health implications [4]. Such studies underscore the potential for vitamin D optimization to serve as a low-cost intervention that might reduce the burden of OM in pediatric populations, particularly in regions where children are at risk of vitamin D deficiency due to limited sunlight exposure or dietary insufficiencies [2,11].

### Study Rationale and Objectives

Despite the well-documented link between vitamin D deficiency and respiratory infections, there remains a notable gap in the literature regarding its specific association with different clinical subtypes of OM. Most previous investigations have focused on the general relationship between vitamin D levels and respiratory infections without stratifying the analysis by the distinct forms of OM, such as ASOM, SOM, or ACOM [22,23]. This is particularly important because each OM subtype may have unique risk factors and may respond differently to interventions, including vitamin D supplementation. The local clinical practice routinely distinguishes these subtypes for diagnostic and therapeutic purposes; therefore, a more detailed analysis could provide insights that would lead to tailored prevention strategies and improved patient outcomes.

The current study is designed to address this gap by evaluating serum vitamin D levels in a cohort of children aged 2–7 years who present with various clinical forms of OM, as well as in a quasi-control group of children without recent OM episodes. Our primary objective is to determine whether lower vitamin D levels are significantly associated with increased frequency and severity of OM episodes, and whether this association differs among the subtypes of OM. Secondary objectives include exploring the potential influence of confounding factors—such as age, residential environment, daycare attendance, and other lifestyle variables—on the relationship between vitamin D status and OM. By employing rigorous statistical methods and adhering to established reporting guidelines, this study seeks to provide clear evidence on the role of vitamin D as a modifiable risk factor in the prevention of recurrent OM.

In summary, the prevalence of OM and its associated complications, combined with the emerging evidence linking vitamin D deficiency to immune dysfunction, call for a focused investigation into how vitamin D status may influence the clinical course of OM. Understanding this relationship is not only important from a clinical perspective but also has broader public health implications. This study aims to build on previous findings by offering a detailed, subtype-specific analysis that could inform future guidelines and interventions, ultimately improving pediatric healthcare outcomes and reducing the burden of OM on affected populations.

## 2. Materials and Methods

### 2.1. Study Design and Participants

This observational study was conducted in accordance with the Strengthening the Reporting of Observational Studies in Epidemiology (STROBE) guidelines. Adhering to these standards ensures transparency and rigor in reporting our methods, findings, and potential limitations [24,25]. The study was performed at a specialized pediatric otolaryngology clinic in Northwest Romania during the winter–spring period (November 2023 to April 2024) when limited sunlight exposure was expected to reduce endogenous vitamin D synthesis.

A total of 118 children, aged between 2 and 7 years, were enrolled in this study. The primary inclusion criterion was the documentation of at least one episode of OM within the preceding six months. In addition, a quasi-control group was established by including 31 children from the same clinical setting who had no recent history of OM or upper respiratory infections. Although this latter subset was considered a quasi-control group, its characterization as truly “healthy” was limited by the pragmatic constraints of recruitment from a clinical setting. This dual-group approach allows for a more robust comparison between the affected and unaffected children, enabling an assessment of whether vitamin D deficiency is specifically associated with OM. Children were further grouped by age into three sub-cohorts—2–3 years (Group 1), 4–5 years (Group 2), and 6–7 years (Group 3)—to allow for the examination of potential age-specific patterns [26].

The exclusion criteria were carefully defined to eliminate potential confounders. Children with a history of severe chronic illnesses—including immunodeficiencies, significant metabolic disorders, or congenital craniofacial malformations—were excluded, as these conditions could independently influence vitamin D metabolism or predispose the children to middle ear pathology. Furthermore, children who were receiving vitamin D supplementation at the time of recruitment were excluded to avoid bias in the measurement of baseline vitamin D status.

**Figure 1:**
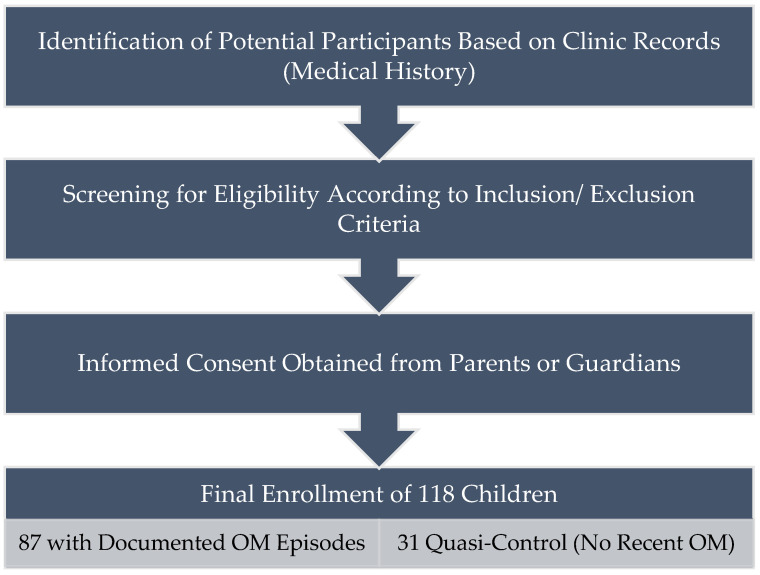
Study Flowchart.

To illustrate the participant recruitment process, we developed a study flowchart (Figure 1). This schematic visualization provides clarity on how participants were selected and how the final study sample was derived.

### 2.2. Data Collection and Vitamin D Assessment

Data collection was conducted using a two-pronged approach—structured parental interviews and a comprehensive review of the medical records. The demographic variables collected included age, sex, and residential environment (categorized as urban or rural). The clinical information encompassed daycare attendance, bottle-feeding practices, and the presence of adenoid hypertrophy—all factors known to potentially influence both vitamin D status and the risk of developing OM.

Environmental factors that might affect susceptibility to OM, such as daycare attendance, passive exposure to cigarette smoke, and the presence of adenoid hypertrophy, were captured through medical records and parental questionnaires. Behavioral and lifestyle factors included the method of early feeding (bottle feeding versus breastfeeding) and household composition, especially the number of siblings, given the potential influence of increased pathogen exposure in larger families. Children who reported any vestibular symptoms or imbalance issues were assessed clinically; however, no participants met the criteria for inclusion based on vestibular disorders, so balance assessments were ultimately not included in the final analysis.

For clinical evaluation, the children underwent otoscopic examinations performed by experienced otolaryngologists. These examinations were used to classify OM into three distinct subtypes based on specific clinical features as follows:

⮚ Acute Suppurative Otitis Media (ASOM): Characterized by a perforated tympanic membrane with purulent discharge.
⮚ Serous Otitis Media (SOM): Identified by the presence of a sterile effusion behind an intact tympanic membrane.
⮚ Acute Congestive Otitis Media (ACOM): Marked by significant inflammation and earache without membrane perforation. This classification is consistent with local clinical practice and facilitates a nuanced understanding of OM’s heterogeneous presentations.

Serum 25-hydroxyvitamin D [25(OH)D] levels were measured using a chemiluminescence immunoassay performed on the ADVIA Centaur XPT analyzer (Siemens Healthineers, Erlangen, Germany). Blood samples were collected from all participants during the study period to minimize seasonal variation. Vitamin D levels were then categorized into three groups—deficient (≤20 ng/mL), insufficient (21–29 ng/mL), and sufficient (≥30 ng/mL)—in accordance with established clinical guidelines [27]. All laboratory assessments were performed in a blinded manner to avoid bias.

According to the manufacturer’s documentation, the ADVIA Centaur XPT Siemens analyzer has an approximate limit of detection (analytical sensitivity) of 4.2 ng/mL. It exhibits an intra-assay coefficient of variation (CV) of about 4.7–5.3% for samples up to 28.2 ng/mL, while its interassay CV ranges from approximately 7.2–11.9% for samples up to 28.2 ng/mL. These performance metrics indicate acceptable precision and reproducibility for routine clinical use.

### 2.3. Statistical Analysis

All statistical analyses were conducted using IBM SPSS Statistics, version 27.0 (IBM Corporation, Armonk, NY, USA). Initially, descriptive statistics were generated to summarize the demographic and clinical characteristics of the study population. Continuous variables, such as serum vitamin D levels, were expressed as means ± standard deviations, while categorical variables were summarized as frequencies and percentages.

To compare the mean vitamin D levels among the different OM subtypes and between the OM cases and the controls, a one-way Analysis of Variance (ANOVA) was conducted. Post hoc comparisons using Tukey’s Honestly Significant Difference (HSD) test were performed to identify specific group differences when overall significance was detected. This method allowed us to determine which groups exhibited statistically significant differences in vitamin D levels.

In order to assess the relationship between the vitamin D levels and the frequency of OM episodes, we employed both simple and multiple linear regression analyses. The simple linear regression model examined vitamin D as a continuous predictor variable, while the multiple regression model adjusted for potential confounders including age, sex, and residential environment. The strength of association was expressed through Pearson’s correlation coefficient and the adjusted R^2^ value.

Furthermore, logistic regression analyses were conducted to estimate the odds of having any form of OM based on vitamin D status. In these models, vitamin D categories (deficient, insufficient, sufficient) were included along with confounding factors such as passive smoking, daycare attendance, and the presence of adenoid hypertrophy. The odds ratios (ORs) with corresponding 95% confidence intervals (CIs) were calculated, with significance set at *p* < 0.05.

Given that the frequency of OM episodes represents count data, Poisson regression was also utilized to estimate the incidence rate ratios (IRRs) for children in the deficient and insufficient vitamin D categories relative to those with sufficient levels. Model fit was assessed by examining deviance and Pearson’s chi-squared statistics, ensuring that the data conformed adequately to the assumptions of the Poisson model.

Although a formal power analysis was not conducted prior to the study initiation due to logistical constraints and the exploratory nature of this research, the sample size of 118 participants was considered sufficient based on previous similar studies in this clinical setting. We acknowledge that the absence of a pre-study power calculation represents a limitation; however, the observed significant associations between vitamin D levels and OM frequency suggest that the study had adequate statistical power to detect meaningful differences. Future studies may benefit from a more formal power analysis to optimize sample size and further validate these findings.

### 2.4. Ethical Considerations

This study was conducted in accordance with the Declaration of Helsinki and received approval from the Ethics Research Committee of the University of Oradea (approval code CEFMF/7, dated 28 March 2024). Informed consent was obtained from the parents or legal guardians of all participating children prior to enrolment. Confidentiality and anonymity were maintained throughout the study, with all the data securely stored and accessible only to authorized personnel.

## 3. Results

### 3.1. Participation Rate and Sample Representativeness

The study enrolled 118 preschool-aged children (2–7 years), achieving an overall participation rate of approximately 67%. This rate was influenced by logistical constraints (such as clinical resources and scheduling) and parental concerns regarding time commitments. Additionally, three children were excluded prior to the study initiation because of anatomical anomalies (e.g., craniofacial malformations), ensuring a consistent sample for analyzing the relationship between vitamin D levels and OM.

### 3.2. Demographic and Clinical Characteristics

Participants were stratified into three age groups to evaluate age-specific variations in vitamin D levels and OM incidence. Overall, 75 children (63.5%) resided in urban settings and 43 (36.5%) resided in rural areas; 48 (40.7%) were female and 70 (59.3%) were male. Table 1 summarizes these demographics by age, sex, and residential environment.

Children with OM were further classified based on otoscopic findings into the following three clinical subtypes (Table 2):

- ASOM (11.86%): Characterized by tympanic membrane perforation and purulent discharge; slightly more common in Groups 1 and 2, with a male predominance (57.14% males vs. 42.86% females).
- SOM (37.29%): The most common subtype, featuring fluid accumulation without overt infection signs; more frequent in urban children (70.45%) and exhibiting a slight male predominance.
- ACOM (24.58%): Marked by mild congestion and earache without membrane perforation; more prevalent among males (72.41% boys vs. 27.59% girls).

### 3.3. Serum Vitamin D Levels

The serum 25-hydroxyvitamin D [25(OH)D] levels were measured in all the participants using a chemiluminescence immunoassay, yielding an overall mean (±SD) of 26.54 ± 10.1 ng/mL. Based on the standard cutoffs, 36.44% of participants were classified as deficient (≤20 ng/mL), 34.74% as insufficient (21–29 ng/mL), and 28.81% as sufficient (≥30 ng/mL). Table 3 presents both the detailed subgroup analysis and the key statistical tests performed.

### 3.4. Integrated Statistical Analyses

To assess the relationship between the vitamin D levels and OM, the following statistical tests were performed:

#### 3.4.1. One-Way ANOVA and Post Hoc Tests

A one-way ANOVA comparing vitamin D levels among the four groups (ASOM, SOM, ACOM, and controls) revealed a highly significant difference (F(3,114) = 82.296, *p* < 0.001) with an effect size (η^2^) of approximately 0.684, indicating that ~68.4% of the variance in vitamin D levels was attributable to group membership. Post hoc Tukey’s HSD tests confirmed that the ASOM group had the lowest vitamin D levels, followed by SOM and ACOM (with no significant difference between the latter two), while the controls had the highest levels.

#### 3.4.2. Chi-Square Analyses

Chi-squared tests were conducted to examine the associations between OM subtypes and categorical variables such as sex, residential environment, bottle-feeding, adenoid hypertrophy, and daycare attendance. No significant associations were found for sex (χ^2^ = 2.965, *p* = 0.397) or residential environment (χ^2^ = 2.045, *p* = 0.563). A borderline trend was observed for bottle-feeding (χ^2^ = 6.930, *p* = 0.074), while adenoid hypertrophy (χ^2^ = 5.821, *p* = 0.121) and daycare attendance (χ^2^ = 5.401, *p* = 0.145) did not reach significance. These results suggest that these categorical variables did not meaningfully differentiate among the OM subtypes in this sample.

#### 3.4.3. Linear Regression Analysis

Simple linear regression showed a strong inverse correlation between the vitamin D levels and the frequency of OM episodes (Pearson’s r = −0.793, *p* < 0.001), with vitamin D explaining approximately 63% of the variance in OM frequency (adjusted R^2^ = 0.629). Specifically, an increase of 1 ng/mL in vitamin D was associated with a reduction of ~0.082 OM episodes over six months. Multiple linear regression, adjusting for age, sex, and environment, confirmed that vitamin D remained the dominant predictor (β ≈ −0.80, *p* < 0.001).

#### 3.4.4. Logistic Regression Analysis

Logistic regression, incorporating vitamin D status along with confounders such as passive smoking, daycare attendance, and adenoid hypertrophy, yielded a strong model fit (Nagelkerke R^2^ ≈ 0.796, classification accuracy = 94.1%). Vitamin D insufficiency was highly significant compared to sufficient status (Exp(B) ≈ 120.74, *p* < 0.001); although estimates for the deficient category were less stable due to sparse data, the overall trend supported the hypothesis.

#### 3.4.5. Poisson Regression Analysis

For the count data on OM episodes, Poisson regression indicated that, relative to sufficient vitamin D levels, children in the deficient category had an incidence rate ratio (IRR) of 13.62, and those in the insufficient category had an IRR of 10.47 (both *p* < 0.001). Model fit statistics (deviance/df = 0.471, Pearson χ^2^/df = 0.559) suggested an acceptable fit despite a slight underdispersion.

### 3.5. Summary of Key Findings

In summary, the analyses consistently demonstrate that low serum vitamin D levels are significantly associated with both the increased risk and higher frequency of OM. Key findings include the following:

**ANOVA:** Significant differences in vitamin D levels among OM subtypes and controls, with the ASOM group exhibiting the most pronounced deficiency.

**Chi-Squared Tests:** No significant associations between OM subtypes and categorical variables (sex, environment), with a borderline trend for bottle-feeding.

**Linear Regression:** A strong inverse relationship between vitamin D levels and OM frequency, with each 1 ng/mL increase in vitamin D reducing OM episodes by ~0.082.

**Logistic Regression:** Vitamin D insufficiency significantly increases the odds of OM (OR ≈ 120.74, *p* < 0.001).

**Poisson Regression:** Children with vitamin D deficiency and insufficiency show significantly higher incidence rates of OM episodes (IRR = 13.62 and 10.47, respectively, *p* < 0.001).

These findings robustly support the hypothesis that low vitamin D status is an independent and modifiable risk factor for recurrent OM in preschool-aged children. The integration of multiple statistical analyses strengthens the evidence that optimizing vitamin D levels may reduce both the occurrence and severity of OM, thereby alleviating its clinical and economic burden.

## 4. Discussion

The current study demonstrates a robust inverse relationship between the serum vitamin D levels and both the risk and frequency of OM in preschool-aged children. Children with lower vitamin D levels, particularly those classified as deficient or insufficient, exhibited a significantly higher frequency of OM episodes. Among the clinical subtypes, ASOM was associated with the most pronounced vitamin D deficiency. These findings indicate that vitamin D, as a modifiable factor, plays a critical role in the pathogenesis of OM and may represent a valuable target for preventive strategies.

A key strength of this study is the comprehensive statistical approach employed. A combination of one-way ANOVA with post hoc analyses, chi-squared tests, linear regression, logistic regression, and Poisson regression was used to assess the association between the vitamin D levels and OM. The one-way ANOVA confirmed significant differences in vitamin D levels among OM subtypes and the control group, with an effect size suggesting that a substantial proportion of the variability in vitamin D levels is explained by group membership. In parallel, the strong inverse correlation identified through linear regression underscores that even modest increases in serum vitamin D are associated with a measurable reduction in OM episodes. Logistic and Poisson regression analyses further substantiate that low vitamin D status significantly elevates both the odds and incidence of OM. Moreover, while categorical variables such as sex, residential environment, bottle-feeding, adenoid hypertrophy, and daycare attendance were examined, these factors did not exhibit significant associations with OM subtypes in the current sample, thereby highlighting the central role of vitamin D.

The findings are consistent with the previous research that has established vitamin D as a critical modulator of immune responses [28,29]. Vitamin D not only contributes to calcium and phosphate homeostasis but also enhances innate immunity through the stimulation of antimicrobial peptides, such as cathelicidin and defensins, which are essential for the initial defense against respiratory pathogens [30,31]. Furthermore, vitamin D modulates adaptive immune responses by regulating cytokine production, potentially mitigating the severity of inflammation during infections. Prior studies have reported associations between vitamin D deficiency and an increased risk of respiratory infections, including OM. The present study extends these observations by providing a detailed, subtype-specific analysis that reveals the most severe vitamin D deficiency in children with ASOM, a finding that carries important clinical implications.

Several biological pathways may account for the association between lower vitamin D levels and elevated risk of OM. One proposed mechanism involves immune modulation, as vitamin D receptors (VDRs) are present on key immune cells such as macrophages and dendritic cells; vitamin D enhances innate immunity while modulating adaptive responses, an effect that potentially reduces inflammation and pathogen burden in the middle ear [32]. Mucosal defense could also play a role, given that adequate vitamin D supports epithelial barrier integrity and triggers antimicrobial peptide production, limiting pathogen colonization and invasion [33]. In addition, vitamin D’s anti-inflammatory effects have been implicated in regulating cytokine profiles by lowering the levels of pro-inflammatory mediators (e.g., IL-6, TNF-α) that are commonly associated with OM pathogenesis [34]. Finally, improvements in Eustachian tube function may arise from sufficient vitamin D, which could favor better muscle tone and mucociliary clearance thus reducing fluid buildup and infection risk [35].

From a clinical and public health perspective, the results have significant implications. In regions characterized by limited sunlight exposure or suboptimal dietary vitamin D intake, routine screening and early supplementation could serve as cost-effective interventions to reduce the burden of OM. Given the high prevalence of OM and its associated complications, including hearing loss and delayed speech development, integrating vitamin D optimization into pediatric preventive strategies may yield substantial benefits. The data suggest that targeting vitamin D deficiency could not only decrease the incidence of OM but also mitigate its severity, thereby reducing the overall clinical and economic burden associated with this common pediatric condition. Raising awareness among parents, caregivers, and healthcare professionals about the role of vitamin D in immune function, alongside the implementation of evidence-based supplementation programs, has the potential to mitigate health disparities—particularly in underserved communities with limited access to nutritious foods or adequate sunlight, a concept that is further supported by research on the role of vitamin D in the aging process [36,37,38]. Moreover, emerging data suggest potential synergistic strategies for mitigating vitamin D deficiency, such as combining vitamin D with other bioactive compounds like those in pomegranates. Recent work has indicated that derivatives like urolithin A might enhance vitamin D receptor activity, amplifying immune responses [39,40,41,42]. Although further study is needed, such integrative nutritional interventions may be particularly advantageous for children susceptible to recurrent infections.

Despite the strengths of the current study, several limitations should be acknowledged. The cross-sectional design precludes definitive conclusions regarding causality; longitudinal studies are required to establish the temporal relationship between vitamin D status and OM. Furthermore, the absence of a formal power analysis, although justified by the exploratory nature of this research, represents a limitation that future studies should address. The quasi-control group, although useful for comparison, was recruited from the same clinical setting and may not fully represent a healthy pediatric population. Additionally, the statistical models, while robust, yielded less stable estimates for the vitamin D deficient category in logistic regression due to sparse data. Future research with larger, more diverse populations and prospective designs will be essential to validate these findings and enhance their generalizability.

In conclusion, the current study provides compelling evidence that low serum vitamin D levels are significantly associated with the increased risk and higher frequency of OM in preschool-aged children. The integration of multiple statistical approaches supports the hypothesis that vitamin D is an independent, modifiable risk factor for recurrent OM. These results underscore the potential clinical utility of vitamin D screening and supplementation as part of a broader strategy to prevent recurrent OM, thereby reducing its impact on child health and healthcare resources. Future investigations should employ longitudinal and interventional study designs to determine whether vitamin D supplementation can effectively reduce the incidence and severity of OM, thereby informing the clinical guidelines and public health policies aimed at optimizing vitamin D status in pediatric populations.

## 5. Conclusions

The findings of the current study demonstrate a significant inverse relationship between serum vitamin D levels and both the incidence and frequency of OM in preschool-aged children. Lower vitamin D levels were notably associated with a higher risk of recurrent OM, particularly in cases of acute suppurative otitis media, where deficiency was most pronounced. These results support the notion that vitamin D deficiency is an independent and modifiable risk factor for OM. Routine screening and targeted supplementation to optimize vitamin D status could therefore serve as effective strategies to reduce the clinical and economic burden of this prevalent pediatric condition [43,44].

Furthermore, the data presented herein provide a robust foundation for the development of prevention strategies centered on improving vitamin D status, especially in regions with limited sunlight exposure and suboptimal dietary intake. While the cross-sectional design of the study limits the ability to draw definitive causal conclusions, the strong associations observed warrant further investigation through longitudinal and interventional studies. Future research should aim to confirm these findings and evaluate the clinical benefits of vitamin D supplementation, ultimately informing updated clinical guidelines and public health policies to enhance pediatric health outcomes.

## Figures and Tables

**Table 1:** Demographic Characteristics by Age, Sex, and Residential Environment.

Age Group	Participants	Female	Male	Urban	Rural
2–3 years	47	19	28	32	15
4–5 years	41	16	25	25	16
6–7 years	30	13	17	18	12
Total	118	48	70	75	43

**Table 2:** Distribution of Otitis Media Subtypes by Age Group and Sex.

OM Type	2–3 y	4–5 y	6–7 y	Female	Male
ASOM	6	5	3	6	8
SOM	11	21	12	19	25
ACOM	14	9	6	8	21
Total (OM)	31	35	21	33	54

**Table 3:** Integrated Summary of Vitamin D Levels and Statistical Analyses.

Subgroup	OM Cases (*n*)	Mean Vit. D in OM Cases (ng/mL) ± SD	Deficiency Rate	Mean Vit. D in Controls (ng/mL) ± SD	Key Statistical Findings
**Age 2–3 years** (Group 1)	31 (66% of group)	18.03 ± 5.28	87% deficient	36.18 ± 4.95	Highest overall deficiency; strong susceptibility to OM
**Age 4–5 years** (Group 2)	35 (85% of group)	22.54 ± 5.00	31% deficient, 63% insufficient	42.50 ± 8.82	94% of OM cases below sufficiency
**Age 6–7 years** (Group 3)	21 (70% of group)	23.90 ± 5.79	24% deficient, 62% insufficient	49.77 ± 5.82	Among OM cases, highest vitamin D levels observed
**Males (OM cases)**	—	20.61 ± 5.72	52% deficient	—	Marginally lower than females
**Females (OM cases)**	—	22.33 ± 5.83	45% deficient	—	Slightly better vitamin D status compared to males
**Urban (OM cases)**	—	20.50 ± 6.17	56% deficient	—	Lower vitamin D levels compared to rural OM cases
**Rural (OM cases)**	—	22.56 ± 4.91	38% deficient	—	Slightly better vitamin D status among rural children
**Overall Statistical Tests:**					ANOVA: F(3,114) = 82.296, *p* < 0.001, η^2^ ≈ 0.684; Chi-square (sex): χ^2^ = 2.965, *p* = 0.397; Linear regression: r = −0.793, R^2^ = 0.629, *p* < 0.001; Logistic: OR ≈ 120.74, *p* < 0.001; Poisson: IRR = 13.62 (deficient), 10.47 (insufficient), *p* < 0.001

## Data Availability

The datasets generated and analyzed during the current study are not publicly available, as the datasets contain secondary outcome parameters which will be used for follow-up research; however, the datasets are available from the corresponding author on reasonable request.

## Abbreviations

The following abbreviations are used in this manuscript:

Abbreviation	Full Term
OM	Otitis Media
ASOM	Acute Suppurative Otitis Media
SOM	Serous Otitis Media
ACOM	Acute Congestive Otitis Media
URTI	Upper Respiratory Tract Infection
ANOVA	Analysis of Variance
SPSS	Statistical Package for the Social Sciences
SD	Standard Deviation
OR	Odds Ratio
IRR	Incidence Rate Ratio
RCT	Randomized Controlled Trial
CI	Confidence Interval
